# Sociedades justas: una nueva visión de la equidad en la salud en la Región de las Américas después de la COVID-19^*^

**DOI:** 10.26633/RPSP.2021.99

**Published:** 2021-08-31

**Authors:** Anna Coates, Arachu Castro, Michael Marmot, Oscar J. Mújica, Gerry Eijkemans, César G. Victora

**Affiliations:** 1 Organización Panamericana de la Salud Washington, D.C. Estados Unidos de América Organización Panamericana de la Salud, Washington, D.C., Estados Unidos de América.; 2 Escuela de Salud Pública y Medicina Tropical de la Universidad Tulane Nueva Orleans Estados Unidos de América Escuela de Salud Pública y Medicina Tropical de la Universidad Tulane, Nueva Orleans, Estados Unidos de América.; 3 Instituto de Equidad en Salud del Colegio Universitario de Londres Londres Reino Unido Instituto de Equidad en Salud del Colegio Universitario de Londres, Londres, Reino Unido.; 4 Universidad Federal de Pelotas Pelotas Brasil Universidad Federal de Pelotas, Pelotas, Brasil.

Los grandes retos para la equidad en la salud en la Región de las Américas, según se detallan en el informe de la Comisión de la Organización Panamericana de la Salud sobre Equidad y Desigualdades en Salud en las Américas (1), dieron el impulso original a este número especial sobre equidad en la salud de la *Revista Panamericana de Salud Pública*. En su informe *Sociedades justas: equidad en la salud y vidas dignas*, se analizó un vasto acervo de evidencia que mostró las abrumadoras desigualdades en la Región relacionadas con tres aspectos: los factores estructurales, las condiciones de la vida cotidiana y la gobernanza para lograr la equidad en la salud (es decir, pasar a la acción).

Este énfasis en la realidad continua de la interrelación entre las inequidades sociales y de salud en la Región de las Américas no es nuevo (2). Sin embargo, desde comienzos del 2020 esta interrelación se ha vuelto más evidente y ha sido exacerbada por la pandemia sin precedentes de la enfermedad por el coronavirus 2019 (COVID-19, por su sigla en inglés), que está poniendo a prueba a los gobiernos, las comunidades, las economías y las personas en formas que nunca hubiésemos imaginado por su alcance e intensidad (3). La crisis está exponiendo las desigualdades subyacentes en la salud y revelando el costo de la falta de acción para abordar esta injusticia social de larga data y, además, la respuesta a la COVID-19 está revirtiendo las mejoras en los indicadores sociales y de salud que se han logrado en los dos últimos decenios (3, 4).

La pandemia pone claramente de manifiesto las desigualdades existentes, así como sus efectos directos e indirectos. Los datos procedentes de diferentes rincones del mundo muestran que el gradiente social para la mortalidad por COVID-19 sigue una trayectoria similar a la del gradiente social para la mortalidad por todas las causas. Se han recopilado datos clave que demuestran las inequidades en los casos de COVID-19, las enfermedades subyacentes y la mortalidad, en países tan diferentes como Brasil y los Estados Unidos de América. El análisis de datos de las encuestas de hogares en Brasil, que se incluye en este número especial, indica que las desigualdades socioeconómicas y de los grupos étnicos están vinculadas al riesgo de infección, y la prevalencia más alta de casos se presenta en grupos indígenas y afrobrasileños en comparación con otros (5). Esto es similar al caso de Estados Unidos, donde el hecho de pasar privaciones y de ser afrodescendiente guarda una fuerte correlación con la mortalidad (6). Por otro lado, la respuesta equitativa a la pandemia en Cuba, que también se incluye en este número, refleja la ventaja de las respuestas nacionales concertadas sustentadas en sistemas sólidos de atención primaria de salud (7). Esto demuestra que, sin voluntad política concertada y esfuerzos dedicados, la riqueza y el estado general de crecimiento económico de un país no constituyen por sí mismos la solución para abordar inequidades en materia de salud. El estudio del caso de Costa Rica, incluido en este número, refuerza el concepto de que, por encima de un umbral, las fortunas económicas no son la clave para el éxito en el campo de la salud (8).

Sin embargo, la historia no acaba con los efectos directos de la COVID-19. También los efectos indirectos están exacerbando las desigualdades existentes en la salud, dado que el acceso a los servicios de salud esenciales se ve amenazado ante la saturación de los sistemas de salud (4). Ya se han observado tasas reducidas de inmunización, a pesar de los grandes esfuerzos que se habían hecho anteriormente para corregir las inequidades (9). El tratamiento de las enfermedades no transmisibles está también afrontando retos, lo que reviste especial importancia para los grupos que se encuentran en un nivel socioeconómico bajo y que enfrentan profundas inequidades en el acceso al dinero y a los recursos que afectan directamente las condiciones de su vida cotidiana. También perjudican a las personas que se enfrentan a la discriminación, como los pueblos indígenas y las poblaciones afrodescendientes, que ya tienen riesgos elevados ante las ENT (10). También se ha expresado preocupación por la falta de acceso a los servicios de salud reproductiva (4, 11, 12) y corren riesgo los servicios para las sobrevivientes de la violencia contra la mujer (11, 13, 14).

Más allá del sector de la salud, los efectos indirectos de la COVID-19 son igualmente perturbadores para las posibilidades de mantener y acelerar los logros en la salud de la población en el futuro con una perspectiva de equidad (3). Las medidas de contención necesarias han repercutido particularmente en los medios de vida de los grupos en situación de vulnerabilidad. Los estratos sociales en mejor posición socioeconómica están resguardados contra las consecuencias más graves de las medidas de confinamiento, puesto que pueden trabajar desde el hogar y vivir en condiciones de menor hacinamiento. Sin embargo, la mayoría de los trabajadores con empleos en condiciones inestables e informales, sin protección social (muchos de los cuales son trabajadores esenciales mal remunerados) no pueden darse estos lujos. Para ellos, adherirse a las medidas de salud pública es excepcionalmente difícil, y las condiciones de hacinamiento incrementan el riesgo de infecciones (y, para un gran número de mujeres y niñas, de ser víctimas de la “pandemia en la sombra” de la violencia contra la mujer) (11). Asimismo, se ven en la obligación de salir del hogar para generar ingresos, resolver la inseguridad alimentaria y satisfacer las necesidades básicas de su familia. La pérdida del empleo y de los ingresos afecta su bienestar y los determinantes sociales de su salud por varios años, dado que se profundizan las inequidades preexistentes y empeoran sus condiciones sociales (15). Diversos grupos están padeciendo la COVID-19 en sí, así como las repercusiones de las medidas de contención, de maneras específicas para su realidad y su cultura que apenas se están empezando a entender. Estas dificultades no solo obstaculizan las respuestas locales y nacionales eficaces, sino que también demuestran la gravedad de los riesgos que corren en su vida, incluso a pesar de que las comunidades y las personas, a falta de otros mecanismos protectores como una protección social adecuada y la salud universal, están promoviendo sus propias formas de resiliencia (10).

Del mismo modo que se va ampliando nuestra comprensión del virus causante de la COVID-19, se están generando datos y análisis para mostrar la plena extensión de las desigualdades con respecto a esta pandemia y sus repercusiones. A medida que intentamos entender sus dimensiones totales en cuanto a la equidad, está saliendo a la luz otra inequidad en la salud: las brechas en nuestro conocimiento y, por lo tanto, en nuestra capacidad de exigir que los gobiernos rindan cuentas sobre la equidad en la salud, dada la falta de datos suficientes y desglosados. En este número se incluye la propuesta de un enfoque con el cual se podría recopilar más información útil para formular políticas orientadas a promover la equidad en la salud (16).

Este número especial refleja el hecho de que la pandemia pone aún más de relieve la urgencia de que haya una acción multisectorial intensificada para la equidad en la salud (17), lo que incluye poner en práctica plenamente las recomendaciones de la Comisión. Esta acción es doble. Por un lado, incluye un espectro amplio de compromisos dentro del sector de la salud, como la atención primaria de salud y la protección social en materia de salud para garantizar la cobertura universal de salud y el acceso universal a la salud dentro de un marco de universalismo proporcional (18). Por el otro, incluye compromisos de trabajar más allá del sector de la salud para abordar los determinantes sociales de la salud, lo que incluye medidas para mejorar las condiciones en las cuales las personas nacen, crecen, viven, trabajan y envejecen, estableciendo un sistema integral de protección social y bienestar basado en la solidaridad y haciendo realidad el potencial redistributivo del gasto social para abordar los determinantes sociales de la salud, tema al que también se refirió el Secretario General de las Naciones Unidas (19).

Se requieren asimismo enfoques nuevos y más profundos para abordar el tema de la equidad en la salud. Nos encontramos en una era de enormes cambios, quizá una de las más significativos y de mayor potencial de nuestra época para destacar y corregir las inequidades en materia de salud de una manera sostenible y transformadora. La atención a estas desigualdades no solo resuena con la realidad que deja al descubierto la actual pandemia de COVID-19, sino también con fenómenos políticos, como el movimiento Black Lives Matter). Además de seguir analizando y adoptando medidas contra las graves inequidades en el acceso al dinero y a los recursos y en las condiciones de vida que afectan la salud, se ha demostrado que la complejidad de abordar las inequidades en materia de salud también requiere diferentes análisis y un énfasis renovado en los factores estructurales. Las conclusiones y recomendaciones de la Comisión ya habían abierto la puerta a estas consideraciones con el reconocimiento explícito de la necesidad de revertir el impacto en la equidad en la salud del colonialismo y el racismo estructural que aún subsisten, así como de la discriminación por razones de género. Ahora tenemos que ir más lejos y lograr un enfoque operativo que vaya más allá de la perspectiva de la vulnerabilidad centrada en grupos de población específicos a una perspectiva que verdaderamente aborde los factores estructurales subyacentes y otros factores sociales y económicos que afectan el acceso a los recursos para la salud, incluidas las medidas explícitas para combatir la discriminación por razones étnicas y de género, y para poner fin al racismo (20).

La variedad de los análisis en los diversos artículos de este número especial refleja esta necesidad de consolidar múltiples enfoques hacia la equidad en la salud. En ellos se pone de relieve desde la necesidad de un enfoque de equidad en los planes nacionales de salud (21), en la infraestructura de salud pública (22) y en el acceso a la tecnología (23) hasta la urgencia de la acción en los determinantes sociales de la salud, pasando por los factores estructurales, incluida la desigualdad en materia de género y el racismo estructural (24, 25), los enfoques interculturales y la medicina tradicional (26). También se demuestra la importancia de los mecanismos de rendición de cuentas, como las funciones de la sociedad civil (27) y la investigación colaborativa (17, 28).

Si aprovechamos la riqueza de estos análisis, tenemos ahora una oportunidad sin precedentes de reconstruir mejor y crear una realidad más inclusiva y equitativa tras la devastación de la COVID-19, una realidad que intente resolver estas complejidades con un compromiso y una finalidad renovados. Varios elementos serán cruciales en nuestra hoja de ruta hacia esta “nueva normalidad”.

Debemos, por ejemplo, considerar los factores estructurales mediante enfoques de derechos humanos y, en particular, mediante una gobernanza incluyente, ya que donde las instituciones no son responsables, transparentes, participativas o coherentes, será mucho menos probable ver el cambio de política que se requiere para lograr la equidad en la salud (29). Esto significa ir más allá de la participación de la comunidad y de la sociedad civil, hacia un modelo de gobernanza incluyente que reajuste las inequidades en el poder y en la opinión para atender los factores estructurales, como el racismo sistémico y la discriminación institucional, entre otros. Diversos grupos tradicionalmente excluidos deben volverse asociados de igual peso en la gobernanza, el liderazgo y la toma de decisiones en un enfoque renovado de la democracia (1). La tercera recomendación general de la Comisión y su subrecomendación sobre la inclusión de los afrodescendientes y las comunidades indígenas en la legislación, el diseño y la provisión de servicios y otras decisiones que afectan sus vidas, preparan el terreno para esta nueva visión radical. En efecto, esto es más pertinente que nunca dada la realidad de la discriminación racial y las disparidades étnicas que dejaron al descubierto la COVID-19 (10) y el movimiento Black Lives Matter. Sin embargo, este enfoque no es exclusivo de la perspectiva de exclusión y discriminación étnica y racial, y puede extenderse para incluir y abordar la discriminación desde otras perspectivas. Estas incluyen, entre otros, el género (en relación con el empoderamiento de las mujeres y las niñas, así como la discriminación que enfrentan los grupos LGBT), los que viven en situaciones de vulnerabilidad socioeconómica, las poblaciones migratorias (30) o quienes están afectados por otras formas de discriminación, por ejemplo, las personas con discapacidad.

La gobernanza incluyente también abarca la rendición de cuentas sobre las acciones y los resultados. En el marco de la recomendación de la Comisión de hacer de la equidad en la salud un indicador clave del desarrollo de la sociedad y de establecer mecanismos de rendición de cuentas, es fundamental que se generen y notifiquen datos desglosados. En consonancia con la primera función esencial de la salud pública —la vigilancia de la salud y el bienestar de la población (31)— y el primer indicador de impacto del actual Plan Estratégico de la OPS (32), “Reducción de las desigualdades en la salud dentro de los países”, es menester que las instituciones inviertan en la capacidad de no solo informar esporádicamente sobre las desigualdades en la salud sino de institucionalizar su seguimiento dentro del análisis de la situación de salud. De esta forma, subsanar las inequidades puede normalizarse como un parámetro del éxito. Además, deben emplearse datos para fundamentar medidas de política a fin de incrementar su impacto potencial. Necesitamos más investigación sobre los aspectos específicos de lo que funciona, así como hacer mejor uso de la información que ya tenemos. La transparencia también constituye la base de una gobernanza incluyente y eficaz. Es necesario poner la evidencia científica a disposición del público, y demostrar cómo se está utilizando esa evidencia sobre las inequidades en la formulación de políticas y en el seguimiento y, quizás aún más importante, señalar dónde están las brechas.

Una nueva visión equitativa para el mundo después de la COVID-19 también requiere reforzar otras formas de trabajar “de otro modo”. Se requiere la cooperación, la colaboración y la gobernanza incluyente a diferentes niveles. Además de trabajar para abordar la equidad dentro de su propia y significativa esfera directa de influencia social, es decir, en las políticas, los programas y los servicios de salud, el sector de la salud tiene que comprometerse a emprender iniciativas intersectoriales con otros asociados gubernamentales en relación con los determinantes sociales de la salud, con base en la comprensión de su importancia para reducir las inequidades en materia de salud. Como lo demuestran la Red de las Américas para la Equidad en Salud (17) y el Movimiento para la Equidad en Salud Sostenible, que se analizan en este número, la colaboración entre las comunidades y los actores a todos los niveles reforzará el impacto potencial en las inequidades en materia de salud en el futuro.

Y, por último, pero con la misma importancia en un mundo cada vez más polarizado, la colaboración a nivel local y mundial entre los países y dentro de ellos para avanzar hacia un modelo más equitativo de la salud y el desarrollo es un imperativo moral. No cabe duda de que esta pandemia ha agudizado las fracturas y creado otras nuevas en nuestra frágil estructura social, pero también nos ha dado un espacio y, esperemos, la voluntad para repararlas. Si la COVID-19 propicia la creación de un modelo renovado de desarrollo basado en un “nuevo pacto social” con compromisos compartidos y cooperación entre los países y las comunidades, tendremos una oportunidad más grande que nunca de corregir las injusticias pasadas y lograr la equidad en la salud en la Región de las Américas.
